# Supplementary material for: Enhancing the Production of Pinene in Escherichia coli by Using a Combination of Shotgun, Product-Tolerance and I-SceI Cleavage Systems
Source: Biology (Basel). 2022 Oct 10;11(10):1484. doi: 10.3390/biology11101484 (PMC9598909; doi:10.3390/biology11101484)
Supplement: Supplementary file 1 [file biology-11-01484-s001.zip › biology-1929473-supplementary.pdf]

Table S1. The primers used in this study.

Name	Sequence (5′-3′)	Purpose
EcoRI-acrB-UF	GACGAATTCgcttaatccaaacgctatttt	PCR for acrB-up homologous arm
BamHI-acrB-UR	GTCGGATCCgtcttaacggctcctgtttaag	
SalI-acrB-DF	GTCGTCGAGatgcctaatttctttatcgatcg	PCR for acrB-down homologous arm
HindIII-acrB-DR	AGTAAGCTTatccgcatcagtaccagactc	
EcoRI-flg-UF	GACGAATTCttgtcggcaactattccaac	PCR for flgF-up homologous arm
BamHI-flg-UR	GTCGGATCC tgagctatcccgtcagcgattag	
SalI-flg-DF	GTCGTCGAGatggatcacgcaatttatac	PCR for flgF-down homologous arm
HindIII-flg-DR	AGTAAGCTTcattacgcgtatacccttcg	
EcoRI-ndk-UF	GACGAATTCcgactttgccgttgcaatca	PCR for ndk-up homologous arm
BamHI-ndk-UR	GTCGGATCCttttacctctgtaaattgttc	
SalI-ndk-DF	GTCGTCGAGatggctattgaacgtactttttc	PCR for ndk-down homologous arm
HindIII-ndk-DR	AGTAAGCTTtaccagccagtgcgtttgcc	
Q-acrBF	atatcgcgcaggttcaggtg	qPCR for acrB
Q-acrBR	agctgttggcctttcaccgg	
Q-flgFF	gcgtaatggcagcattcagg	qPCR for flgF
Q-flgFR	gattgagcgccgagattgtg	
Q-ndkF	ttccatcatcaaaccgaacg	qPCR for ndk
Q-ndkR	aaccagaccatcaaagaacg	
Q-cysGF	ttgtcggcggtggtgatgtc	qPCR for cysG
Q-cysGR	atgcggtgaactgtggaataaacg	

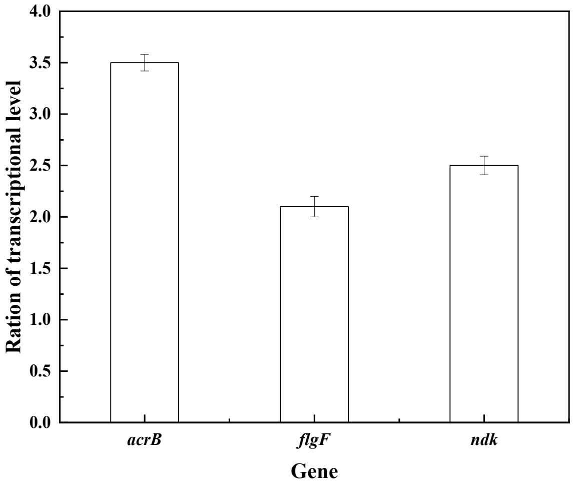

Figure S1. The relative transcriptional levels of the *acrB*, *flgF* and *ndk* genes in *E. coli* MG1655B, MG1655BF and MG1655BFN compared with those in *E. coli* MG1655, respectively. The total RNA from *E. coli* cells for 30 h in shake flasks was isolated using an RNA extraction kit (Tiangen, China), RNase-free gDNaseI was treated during the isolation procedure. The first-strand cDNA was synthesized using an All-in-OneTM First-Strand cDNA Synthesis kit (GeneCopoeia, Guangzhou, China). Quantitative real-time PCR (qRT-PCR) was performed with the All-in-OneTM qPCR Mix kit (GeneCopoeia) by an iCycler iQ5 Real Time PCR system (Bio-Rad Laboratories, California, USA). follows: 95 °C for 10 min, followed by 45 cycles of denaturation at 95 °C for 10 s, annealing at 60 °C for 20 s, and extension at 72 °C for 15 s. The expression levels were analyzed by the $2^{-\triangle\triangle Ct}$ method described by Livak and Schmittgen [1] and normalized by *cysG* gene expression.

Supple

P37-S-Kan-S-P37 sequence (5′-3′)

cttacatgaaaaaggttcttgacattttaaatccatgtggtatatgtcatttttctatttcggaattaaggaggtaata

aattaggga

P37 promoter S

site

Taacagggtaattctgatcaagagacaggatgaggatcgtttcgcatgattgaacaagatggattgcacgcag

gttctccggccgcttgggtggagaggctattcggctatgactgggcacaacagacaatcggctgctctgatgcc

gccgtgttccggctgtcagcgcaggggcgcccggttctttttgtcaagaccgacctgtccggtgccctgaatgaa

ctccaagacgaggcagcgcggctatcgtggctggccacgacgggcgttccttgcgcagctgtgctcgacgttgt

cactgaagcgggaagggactggctgctattgggcgaagtgccggggcaggatctcctgtcatctcaccttgctc

ctgccgagaaagtatccatcatggctgatgcaatgcggcggctgcatacgcttgatccggctacctgcccattcg

accaccaagcgaaacatcgcatcgagcgagcacgtactcggatggaagccggtcttgtcgatcaggatgatct

ggacgaagagcatcaggggctcgcgccagccgaactgttcgccaggctcaaggcgcggatgcccgacggcg

aggatctcgtcgtgacccatggcgatgcctgcttgccgaatatcatggtggaaaatggccgcttttctggattcat

cgactgtggccggctgggtgtggcggaccgctatcaggacatagcgttggctacccgtgatattgctgaagag

cttggcggcgaatgggctgaccgcttcctcgtgctttacggtatcgccgctcccgattcgcagcgcatcgccttct

atcgccttcttgacgagttcttctgaccggaattgccagctggggcgccctctggtaaggttgggaagccctgca

aagtaaactggatggctttcttgccgccaaggatctgatggcgcaggggatcaagatagggataacagggtaa

tcttacatg

Kan S site

Aaaaaggttcttgacattttaaatccatgtggtatatgtcatttttctatttcggaattaaggaggtaataaat

P37 promoter
